# Performance of standardized patient reported outcomes developed for spondyloarthritis in primary and concomitant forms of fibromyalgia

**DOI:** 10.1186/s13075-024-03365-y

**Published:** 2024-07-26

**Authors:** Styliani Tsiami, Piet Dukatz, Maria Gkelaki, Philipp Sewerin, Uta Kiltz, Xenofon Baraliakos

**Affiliations:** Rheumazentrum Ruhrgebiet, Ruhr Universität Bochum, Claudiusstr.45, 44649 Herne, Germany

**Keywords:** Axial spondyloarthritis, Psoriatic arthritis, Fibromyalgia, Widespread pain, Disease activity

## Abstract

**Background:**

In spondyloarthritides (SpA) and fibromyalgia (FM), patients suffer from generalized pain. The impact of FM on PRO validated in SpA has not been systematically studied.

**Objective:**

Study the performance of PROs developed for SpA in patients with primary (p) FM without chronic inflammatory-rheumatic disease vs. SpA without and with concomitant (c) FM.

**Methods:**

Patients with pFM, axSpA or PsA and indication for treatment adaptation were prospectively included. Standardized PROs were assessed: BASDAI, ASDAS-CRP, DAPSA, patient´s global assessment, BASFI, LEI, MASES, SPARCC Enthesitis Score and FIQ.

**Results:**

300 patients were included (100/diagnosis). More males were found in axSpA vs. PsA and pFM group (67, 33 and 2/100, respectively), while 12 axSpA (axSpA+) and 16 PsA (PsA+) patients had cFM. pFM patients showed significantly higher scores in all assessments vs. axSpA or PsA, with exception of ASDAS-CRP (3.3 ± 0.6 in FM vs. 3.1 ± 1.0 in axSpA) and duration of low lumbar morning stiffness. Similar results were also found in the subanalysis of female patients only. In addition, patients with axSpA + or PsA + showed no differences to patients with pFM, while significantly higher scores were found for FM, axSpA + and PsA + for almost all FIQ items compared to axSpA- or PsA-.

**Conclusions:**

PROs originally developed for axSpA or PsA need to be interpreted differently in the presence or absence of cFM. ASDAS-CRP and duration of lumbar morning stiffness were not affected by cFM. FM-specific questionnaires also showed high scores in patients with SpA with cFM but not in those without.

## Introduction

The term spondyloarthritis (SpA) encompasses a group of inflammatory rheumatic diseases with some clinical and genetic similarities, particularly characterized by inflammation of the axial skeleton, peripheral joints and tendon insertions, and association with the major histocompatibility complex (MHC) class I human leukocyte antigen (HLA)-B27 [1]. SpA includes both axial (axSpA) and peripheral forms of the disease, the main representative of the latter being psoriatic arthritis (PsA). With a prevalence of about 0.8%, axSpA is among the most frequent rheumatic diseases [2] and predominantly presents as inflammatory back pain [3], starting in the third decade of life [1], leading to symptoms like stiffness and decreased function and mobility due to osteoproliferative changes in the sacroiliac joints (SIJ) and the spine [4]. PsA, affecting about 0.3% of the population [2, 5] is associated with cutaneous psoriasis and features asymmetric oligoarthritis, dactylitis, and enthesitis [6, 7].

Fibromyalgia (FM), a non-inflammatory chronic musculoskeletal disease characterized by chronic widespread pain and fatigue, affects about 2% of the population, predominantly women [8]. The most characteristic symptom of FM is widespread pain [9]. However, patients with FM may also present with symptoms overlapping with axSpA and PsA, complicating differentiation [10, 11]. In addition, evidence suggests FM occurs frequently [12] with rheumatic and musculoskeletal diseases (RMD), impacting patient-reported outcomes (PROs) in axSpA und PsA [13].

Patient-reported outcomes (PROs) are crucial tools for assessment of several aspects of SpA [14], similar to other inflammatory rheumatic diseases. In routine clinical practice these assessments are well implemented and used on regular basis. Nevertheless, several studies indicate that cFM in patients with axSpA and PsA [15, 16] affects PROs [17–20], yet the extent of this impact remains unclear [20–22].

In the present study, we aimed to prospectively assess the influence of pFM on the performance of disease-specific and general assessments and PROs of axSpA and PsA, both with and without cFM.

## Patients and methods

### Study population

In a prospective, comparative, cross-sectional study, male and female patients ≥18 years of age and a confirmed diagnosis of axSpA or PsA, with (axSpA + and PsA+) or without (axSpA- and PsA-) cFM or patients with pFM, all confirmed by experienced rheumatologists, and an indication for a treatment adaptation (escalation or change of already existing treatment) were included after informed consent between May 2019 and August 2020. Patients with FM had to fulfill the 2016 ACR diagnostic criteria [23], while patients with axSpA and PsA had to have the respective diagnosis of axSpA or PsA given by the treating rheumatologist and also fulfill the ASAS [24] and CASPAR criteria [25], respectively. A total population of 300 patients was planned and approved by the ethic committee of the Ruhr Universität Bochum (18-6607-BR). Patients were included consecutively, allocated 1:1:1 to each group until 100 patients were reached in each group.

### Assessments

Standardized assessment tools according to the respective diagnosis were applied to patients with axSpA (numerical pain rating scale (NRS pain) [26], Bath Ankylosing Spondylitis Disease Activity Index (BASDAI) [27], Ankylosing Spondylitis Disease Activity Score (ASDAS-CRP) [28], Bath Ankylosing Spondylitis Functioning Index (BASFI) [29], Maastricht Ankylosing Spondylitis Enthesitis Score (MASES) [30], Leeds Enthesitis Index (LEI) and Spondyloarthritis Research Consortium of Canada scoring system (SPARCC) [31]) or PsA (NRS pain, Disease Activity score for Psoriatic Arthritis (DAPSA) [32], MASES, LEI, and SPARCC). In addition, patients from both groups also filled out the Fibromyalgia Impact Questionnaire (FIQ) [33]. In patients with pFM, all assessments (NRS pain, BASDAI, ASDAS-CRP, BASFI, DAPSA, SPARCC, LEI, MASES and FIQ) were applied.

### Statistical analysis

Descriptive data are shown as absolute numbers and percentages for qualitative variables. Continuous variables are shown as mean values ± standard deviations. The Mann-Whitney-U test was used for calculation of categorical variables and the t-test was used for continuous variables. A p-value < 0.05 was considered statistically significant. Statistical analysis was performed with SPSS v. 28.

## Results

### Patients’ characteristics

The baseline demographics are shown in Table 1. In brief, the mean age was 52.9 ± 14.1 years in axSpA, 56.4 ± 12.8 years in PsA and 56.4 ± 10.2 years in FM. More male patients were found in the axSpA group (67/100 patients), while the PsA group and the FM group included more female patients (67/100 and 98/100, respectively).

**Table 1:** Baseline characteristics of all patients related to the diagnoses pFM or axSpA or PsA with cFM or without FM

	FM	axSpA-	*p*-value axSpA- vs. FM	axSpA+	*p*-value axSpA + vs. FM	PsA-	*p*-value PsA- vs. FM	PsA+	*p*-value PsA + vs. FM
Age	56.4 ± 10.2	53.5 ± 14.2	0.086	48.7 ± 13.5	0.026	56.0 ± 13.2	< 0.001	58.5 ± 10.4	0.962
Male	2%	67%	< 0.001	0%	---	38%	< 0.001	6%	---
HLAB27 pos.	0%	84%	---	71%	---	16%	---	50%	---
CRP (mg/dl)	0.3 ± 0.5	1.1 ± 2.1	0.001	0.5 ± 0.3	0.035	1.4 ± 3.0	0.001	0.5 ± 0.5	0.031
NRS pain	7.5 ± 1.6	6.1 ± 2.4	< 0.001	7.7 ± 1.8	0.768	6.4 ± 2.2	< 0.001	7.3 ± 1.1	0.223

Fibromyalgia (FM); axial spondyloarthritis (axSpA); psoriatic arthritis (PsA)

axSpA-: axSpA without concomitant FM (cFM), axSpA+: axSpA with cFM, PsA-: PsA without cFM, PsA+: PsA with cFM

HLA B27: human leucocyte antigen B27; CRP: C-reactive protein; NRS: numerical rating scale

### Influence of primary FM on disease-specific and general assessments and PROs of axSpA and PsA

Patients with pFM showed significantly higher scores in almost all assessments as compared to patients with axSpA- or PsA- (Table 2). The only assessments with no significant (but numerical) differences were ASDAS-CRP (3.3 ± 0.6 in pFM vs. 3.1 ± 1.0 in axSpA-) and the duration of morning stiffness in the lower back (question 6 of the BASDAI), with 4.7 ± 2.2 in pFM vs. 4.6 ± 2.7 in axSpA-. The detailed results of all analyses are shown in Table 2.

**Table 2:** Baseline scores of different items of all patients related to the diagnoses pFM or axSpA or PsA with cFM or without FM

	FM	axSpA-	*p*-value axSpA- vs. FM	axSpA+	*p*-value axSpA + vs. FM	PsA-	*p*-value PsA- vs. FM	PsA+	*p*-value PsA + vs. FM
BADSAI	6.9 ± 1.4	5.2 ± 2.0	< 0.001	6.9 ± 1.4	0.858	---	---	---	---
ASDAS-CRP	3.3 ± 0.6	3.1 ± 1.0	0.086	3.7 ± 1.2	0.208	---	---	---	---
BASFI	6.4 ± 2.1	5.4 ± 2.5	0.005	7.1 ± 1.8	0.41	---	---	---	---
DAPSA	43.0 ± 17.8	---	---	---	---	32.0 ± 18.6	< 0.001	46.5 ± 19.7	0.37
FIQ	68.5 ± 13.5	53.9 ± 21.2	< 0.001	72.3 ± 13.7	0.352	57.2 ± 18.3	< 0.001	68.5 ± 11.6	0.978
LEI	4.0 ± 1.6	1.5 ± 1.7	< 0.001	3.3 ± 1.4	0.179	2.4 ± 2.0	< 0.001	3.6 ± 2.0	0.625
MASES	8.6 ± 3.0	3.4 ± 3.3	< 0.001	8.2 ± 2.9	0.642	4.2 ± 3.6	< 0.001	7.1 ± 3.6	0.101
SPARCC	9.4 ± 3.4	3.5 ± 3.4	< 0.001	7.7 ± 3.8	0.139	5.1 ± 3.7	< 0.001	8.1 ± 3.9	0.412

Fibromyalgia (FM); axial spondyloarthritis (axSpA); psoriatic arthritis (PsA), BASDAI: Bath Ankylosing Spondylitis Disease Activity Index BASFI: Bath Ankylosing Spondylitis Functioning Index

axSpA-: axSpA without concomitant FM (cFM), axSpA+: axSpA with cFM, PsA-: PsA without cFM, PsA+: PsA with cFM

### Subgroup analysis for patients with axSpA or PsA with concomitant FM

Overall, 12/100 and 16/100 patients were diagnosed with axSpA + and PsA+, respectively. Patients with axSpA + showed a significantly lower mean age (48.7 ± 13.0 years) as the mean age of the entire axSpA group (52.9 ± 14.1 years), while the mean age of PsA + patients was not different (58.5 ± 10.4 years) to the mean in the PsA group (56.4 ± 12.8 years) (Table 1).

Patients with axSpA + and PsA + showed significantly higher scores in almost all assessments as compared to patients with axSpA- and PsA-, with exception of ASDAS-CRP (3.7 ± 1.2) and the duration of morning stiffness in the lower back (question 6 of the BASDAI) (Table 2). The results of patients with axSpA + and PsA + were similar to those obtained when these scores were applied to patients with pFM (Table 2).

### Subgroup analysis for female patients only

In the analysis of female patients only, female axSpA + and PsA + patients had significantly higher scores in almost all assessments as compared to axSpA- and PsA- patients (Table 3), while the same was found in the comparison between female patients with pFM vs. axSpA- or PsA- (Table 3). Female patients with axSpA + or PsA + showed no differences to patients with pFM.

**Table 3:** Baseline scores of different items of all female patients related to the diagnoses pFM or axSpA or PsA with cFM or without FM

	FM	axSpA-	*p*-value to FM	axSpA+	*p*-value to FM	*p*-value within axSpA	PsA-	*p*-value to FM	PsA+	*p*-value to FM	*p*-value within PsA
BADSAI	6.9 ± 1.4	5.3 ± 1.9	< 0.001	6.9 ± 1.4	0.886	0.009	---	---	---	---	
ASDAS-CRP	3.3 ± 0.6	2.9 ± 0.8	0.019	3.7 ± 1.2	0.206	0.019	---	---	---	---	
BASFI	6.4 ± 2.1	4.8 ± 2.5	0.002	7.1 ± 1.8	0.418	0.015	---	---	---	---	
DAPSA	42.8 ± 17.4	---	---	---	---		34.8 ± 17.7	0.013	47.6 ± 19.9	0.238	0.023
FIQ	68.5 ± 13.6	51.9 ± 21.7	< 0.001	72.3 ± 13.7	0.362	0.005	56.6 ± 16.8	< 0.001	67.6 ± 11.3	0.725	0.01
LEI	3.9 ± 1.6	2.1 ± 1.8	< 0.001	3.3 ± 1.4	0.195	0.031	2.9 ± 1.9	0.001	3.5 ± 2.0	0.435	0.294
MASES	8.6 ± 3.0	3.9 ± 2.9	< 0.001	8.2 ± 2.9	0.639	< 0.001	5.2 ± 3.6	< 0.001	6.7 ± 3.3	0.04	0.117
SPARCC	9.3 ± 3.3	4.6 ± 3.3	< 0.001	7.7 ± 3.8	0.146	0.032	6.3 ± 3.4	< 0.001	7.9 ± 4.0	0.328	0.075

### Comparison of fibromyalgia assessments among patients with fibromyalgia and spondyloarthritis

The analysis of the total FIQ but also of its single items revealed similar results as the analysis of the axSpA-related and PsA-related assessments (Table 2). Patients with axSpA + or PsA + showed no differences to patients with pFM for all FIQ items with exception of the item “walk several blocks” (Table 4). On the other hand, significant differences in the FIQ results were found between axSpA + and axSpA- as well as between PsA + and PsA- patients (Table 4).

**Table 4:** Level of the Fibromyalgia Impact Questionaire (FIQ) in patients with fibromyalgia (FM) with cFM) and without pFM axial axSpA or PsA

	FM	axSpA -	*p*-value axSpA- vs. FM	axSpA +	*p*-value axSpA + vs. FM	PsA-	*p*-value PsA- vs. FM	PsA+	*p*-value PsA + vs. FM
FIQ total Score	68.5 ± 13.5	53.9 ± 21.1	**< 0.001**	72.3 ± 13.7	**0.35**	57.2 ± 18.3	**< 0.001**	68.5 ± 11.6	0.98
Shopping	1.4 ± 0.8	0.99 ± 0.943	**< 0.001**	1.8 ± 0.9	**0.304**	1.2 ± 1.0	0.057	1.5 ± 0.7	0.74
Laundry	1.0 ± 0.8	0.91 ± 1.048	0.216	1.3 ± 1.1	**0.449**	0.9 ± 1.1	0.083	1.1 ± 0.9	0.803
Cook	1.4 ± 1.3	0.91 ± 0.957	**0.005**	1.5 ± 0.9	**0.555**	1.0 ± 1.0	**0.024**	1.4 ± 1.0	0.564
Wash dishes	1.6 ± 1.0	0.96 ± 1.089	**< 0.001**	1.8 ± 1.0	**0.492**	1.2 ± 1.1	**0.006**	1.6 ± 0.8	0.901
Vacuum	1.8 ± 0.8	1.26 ± 1.163	**< 0.001**	2.0 ± 0.7	**0.475**	1.4 ± 1.0	**0.01**	1.8 ± 0.9	0.782
Make beds	1.7 ± 0.9	1.14 ± 1.073	**< 0.001**	2.0 ± 0.9	**0.306**	1.3 ± 1.1	**0.008**	1.6 ± 0.8	0.752
Walk several blocks	1.6 ± 0.8	1.28 ± 1.102	**0.018**	2.4 ± 0.7	**0.007**	1.3 ± 1.1	**0.041**	2.1 ± 0.7	0.062
Visit friends	1.6 ± 0.9	1.12 ± 1.034	**0.002**	1.8 ± 1.0	**0.421**	1.2 ± 1.1	**0.016**	1.7 ± 0.8	0.787
Yard work	2.5 ± 0.6	1.92 ± 1.08	**0.001**	2.4 ± 0.7	**0.904**	2.0 ± 1.0	**0.007**	2.7 ± 0.6	0.252
Drive a car	1.4 ± 0.9	1.06 ± 1.144	**0.014**	1.6 ± 1.0	**0.743**	1.1 ± 1.1	**0.02**	1.3 ± 1.0	0.647
Climb stairs	1.6 ± 0.7	1.11 ± 0.982	**< 0.001**	1.7 ± 0.7	**0.766**	1.4 ± 0.9	0.057	1.9 ± 0.9	0.072
Count good days	1.6 ± 1.6	2.49 ± 2.223	**0.005**	1.3 ± 1.0	**0.902**	2.6 ± 2.3	**0.001**	1.5 ± 1.7	0.894
Count miss work days	4.2 ± 2.5	3.2 ± 3.019	**0.024**	4.7 ± 2.4	**0.527**	3.8 ± 2.7	0.286	4.2 ± 2.8	0.936
Affected by pain	7.5 ± 2.0	6.34 ± 2.668	**0.003**	7.9 ± 1.4	**0.631**	6.7 ± 2.3	**0.008**	7.4 ± 1.7	0.513
Severe pain	7.8 ± 1.4	6.3 ± 2.4	**< 0.001**	7.7 ± 1.9	**0.81**	6.8 ± 2.1	**< 0.001**	7.3 ± 1.4	**0.08**
Fatigue	7.6 ± 2.0	6.1 ± 2.7	**< 0.001**	8.5 ± 1.4	**0.09**	6.2 ± 2.8	**< 0.001**	8.5 ± 1.6	**0.06**
Feeling after getting up	8.1 ± 1.9	6.69 ± 2.534	**< 0.001**	8.8 ± 1.5	**0.167**	6.3 ± 2.7	**< 0.001**	8.5 ± 1.8	0.555
Stiffness	7.1 ± 1.9	6 ± 2.491	**0.004**	7.4 ± 2.4	**0.352**	6.1 ± 2.7	**0.026**	7.7 ± 2.2	0.147
Anxiety	5.5 ± 2.6	4.0 ± 3.0	**< 0.001**	5.9 ± 3.5	**0.41**	4.2 ± 2.9	**0.003**	4.9 ± 3.0	0.58
Depression	5.8 ± 2.4	4.11 ± 3.087	**< 0.001**	5.0 ± 3.3	**0.474**	5.0 ± 3.1	0.102	5.3 ± 3.3	0.681

## Discussion

To our knowledge, this is the first prospective study that examines the performance of standardized patient-reported outcomes (PROs) for both axial spondyloarthritis (axSpA) and psoriatic arthritis (PsA) in patients with primary fibromyalgia (pFM). Additionally, it compares these outcomes to the subgroups of patients with axSpA and PsA with and without concomitant fibromyalgia (FM) in a real-life setting.

Recent studies have shown that FM is common not only in the general population [19] but also appears frequently as a ‘secondary’ FM in chronic inflammatory rheumatic diseases [34] particularly in axSpA [19] and PsA [35], the latter showing prevalences of around 20% of cFM in the examined cohorts. Objective evaluations, such as ultrasound examinations, showed recently that PsA patients present with a higher number of involved entheses and specific patterns of entheseal involvement than patients with FM [36]. However, it remains unclear if these evaluations can distinguish between polyenthesitis and FM in the same patient.

Previous data comparing the performance of some PROs before and after biologic drug treatment have indicated that cFM negatively impacts treatment response in both axSpA [17, 21] and PsA [37]. These studies, which were retrospective and based on existing patient´s records, highlighted the need for a prospective evaluation of whether clinical indices and especially the PROs are influenced by cFM and, subsequently, how these data compare in patients with pFM diagnosis without any other RMD condition.

Our results, which included the most frequently used PROs for both axSpA and PsA, confirm FM as an important comorbidity in these primary chronic inflammatory diagnoses and show that almost all of them well-established PROs used in axSpA and PsA need to be interpreted differently in the presence or absence of cFM both in daily practice but also in the setting of clinical studies.

Importantly we report that especially when it comes to PROs originally developed for assessment of disease activity in both axSpA and PsA, such results need to be interpreted differently when cFM is present. While ASDAS-CRP and the duration but not the severity of morning low back pain stiffness were not significantly affected by cFM, the BASDAI did show such influence. This discrepancy can be attributed to the nature of these assessments: BASDAI relies on subjective patient-reported outcomes, which are influenced by FM, whereas ASDAS-CRP includes an objective component (CRP levels), providing a more balanced assessment of inflammatory disease activity.

In more detail, the analysis of all individual questions of PROs showed that FM negatively affects all areas of patients’ health and daily life. Specifically, fatigue (question 1 of the BASDAI), pain in the entheses and joints, back pain and physical function were significantly worsened by the co-existence of fibromyalgia.

Furthermore, FM-specific questionnaires also showed high scores in patients with SpA with cFM but not in those without. Since widespread pain due to cFM is unlikely to be affected by anti-inflammatory treatment [38], it seems appropriate to capture this comorbidity prior to the start of therapy in patients with no evidence of inflammation but high levels of diffuse pain, including unclear signs of enthesitis [36].

A limitation of our study is the small sample size of axSpA + and PsA+ (12 and 18 respectively), which might be considered too small to draw definitive conclusions. However, these numbers were dependent based on the prevalence of cFM in axSpA and PsA patients, and expected based on the existing literature [39–41]. Previous studies [17, 21, 22] have used the FiRST questionnaire to screen patients for FM at study inclusion, resulting to somewhat higher prevalences of patients affected by FM, while our study used diagnoses made by experienced rheumatologists and took also into account the revised ACR 2016 criteria, which involve a comprehensive clinical evaluation process that includes both a detailed history and physical examination. That may also explain the small differences to previous results. Furthermore, we did not collect information on possible treatment specifically for FM symptoms. However, since all patients included in this study were admitted due to an exacerbation of their primary inflammatory disease or due to exacerbation of their FM symptoms and were therefore considered ‘active’, we believe that the possible bias for interpretation of the questionnaires is rather minor.

The strengths of our study include its prospective design and the inclusion of a representative number of SpA and FM patients without any exclusion that could bias outcomes. Furthermore, the high level of clinical activity symptoms in the patients makes our results more relevant to real-life clinical context regarding the influence of cFM on treatment decisions.

## Conclusion

In conclusion, the data of the present prospective study underscore the importance of using both subjective and objective measures in disease activity assessment, especially in patients with overlapping conditions like FM and SpA. More research is needed to determine the best approaches for interpreting PROs in these patients. Finally, it is reassuring that ASDAS-CRP seems to not be affected by cFM, while BASDAI does not seem to have such ability – something that needs to be considered also for choosing primary outcomes in clinical studies [42].

## Data Availability

The datasets used and/or analysed during the current study are available from the corresponding author on reasonable request.

## Abbreviations

ASDAS-CRP: Ankylosing Spondylitis Disease Activity Score
axSpA: axial spondyloarthritis
BASDAI: Bath Ankylosing Spondylitis Disease Activity Index
BASFI: Bath Ankylosing Spondylitis Functioning Index
cFM: concomitant fibromyalgia
DAPSA: Disease Activity score for Psoriatic Arthritis
FIQ: Fibromyalgia Impact Questionnaire
FM: fibromyalgia
HLA-B27: human leukocyte antigen- B27
LEI: Leeds Enthesitis Index
MASES: Maastricht Ankylosing Spondylitis Enthesitis Score
MHC: major histocompatibility complex class I
MRI: magnetic resonance imaging
NRS pain: numerical pain rating scale
pFM: primary fibromyalgia
PROs: Patient-reported outcomes
PsA: psoriatic arthritis
RMD: rheumatic and musculoskeletal diseases
SIJ: sacroiliac joints
SpA: spondyloarthritis
SPARCC: Spondyloarthritis Research Consortium of Canada scoring system

## References

[1] Sieper J, Poddubnyy D. Axial spondyloarthritis. Lancet (London England). 2017;390(10089):73–84. 10.1016/S0140-6736(16)31591-4. 28110981 10.1016/S0140-6736(16)31591-4

[2] Zink A, Albrecht K. How frequent are musculoskeletal diseases in Germany? Z Rheumatol. 2016;75(4):346–53. 10.1007/s00393-016-0094-2. 27142379 10.1007/s00393-016-0094-2

[3] Baraliakos X, Tsiami S, Redeker I, et al. Early recognition of patients with axial spondyloarthritis - evaluation of referral strategies in primary care. Revmatol. 2020;59(12):3845–52. 10.1093/rheumatology/keaa212. 10.1093/rheumatology/keaa212 32472689

[4] Essers I, Boonen A, Busch M, et al. Fluctuations in patient reported disease activity, pain and global being in patients with ankylosing spondylitis. Rheumatology (Oxford). 2016;55(11):2014–22. 10.1093/RHEUMATOLOGY/KEW303. 27520798 10.1093/RHEUMATOLOGY/KEW303

[5] Alamanos Y, Voulgari PV, Drosos AA. Incidence and prevalence of psoriatic arthritis: A systematic review. *J Rheumatol*. 2008;35(7):1354–1358. Accessed January 14, 2022. https://pubmed.ncbi.nlm.nih.gov/18464305/. 18464305

[6] Mease PJ, Gladman DD, Papp KA, et al. Prevalence of rheumatologist-diagnosed psoriatic arthritis in patients with psoriasis in European/North American dermatology clinics. J Am Acad Dermatol. 2013;69(5):729–35. 10.1016/J.JAAD.2013.07.023. 23981683 10.1016/J.JAAD.2013.07.023

[7] Kavanaugh A, Helliwell P, Ritchlin CT. Psoriatic arthritis and Burden of Disease: patient perspectives from the Population-based multinational Assessment of Psoriasis and Psoriatic Arthritis (MAPP) Survey. Rheumatol Ther. 2016;3(1):91–102. 10.1007/S40744-016-0029-Z. 27747516 10.1007/S40744-016-0029-Z PMC4999579

[8] Bellato E, Marini E, Castoldi F, et al. Fibromyalgia syndrome: etiology, pathogenesis, diagnosis, and treatment. Pain Res Treat. 2012;2012. 10.1155/2012/426130. 10.1155/2012/426130 PMC3503476 23213512

[9] Bennett RM, Russell J, Cappelleri JC, Bushmakin AG, Zlateva G, Sadosky A. Identification of symptom and functional domains that fibromyalgia patients would like to see improved: a cluster analysis. BMC Musculoskelet Disord. 2010;11. 10.1186/1471-2474-11-134. 10.1186/1471-2474-11-134 PMC2908076 20584327

[10] Góes SM, Leite N, Shay BL, Homann D, Stefanello JMF, Rodacki ALF. Functional capacity, muscle strength and falls in women with fibromyalgia. Clin Biomech (Bristol Avon). 2012;27(6):578–83. 10.1016/J.CLINBIOMECH.2011.12.009. 22230426 10.1016/J.CLINBIOMECH.2011.12.009

[11] Jones SD, Koh WH, Steiner A, Garrett SL, Calin A. Fatigue in ankylosing spondylitis: Its prevalence and relationship to disease activity, sleep, and other factors. *J Rheumatol*. 1996;23(3):487–490. Accessed April 30, 2022. https://pubmed.ncbi.nlm.nih.gov/8832988/. 8832988

[12] Fan A, Pereira B, Tournadre A, et al. Frequency of concomitant fibromyalgia in rheumatic diseases: monocentric study of 691 patients. Semin Arthritis Rheum. 2017;47(1):129–32. 10.1016/J.SEMARTHRIT.2017.01.005. 28216193 10.1016/J.SEMARTHRIT.2017.01.005

[13] Alunno A, Carubbi F, Stones S, Gerli R, Giacomelli R, Baraliakos X. The impact of Fibromyalgia in Spondyloarthritis: from classification criteria to Outcome measures. Front Med. 2018;5(OCT). 10.3389/FMED.2018.00290. 10.3389/fmed.2018.00290 PMC6207601 30406105

[14] Navarro-Compán V, Boel A, Boonen A, et al. Instrument selection for the ASAS core outcome set for axial spondyloarthritis. Ann Rheum Dis Published Online. 2022. 10.1136/ANNRHEUMDIS-2022-222747. 10.1136/ANNRHEUMDIS-2022-222747 35680390

[15] Lubrano E, Scriffignano S, Morelli R, Perrotta FM. Assessment of widespread and Extraarticular Pain in Psoriatic Arthritis: a case-control study. J Rheumatol. 2021;48(9):1405–9. 10.3899/JRHEUM.201163. 33452167 10.3899/JRHEUM.201163

[16] Brikman S, Furer V, Wollman J, et al. The Effect of the Presence of Fibromyalgia on Common Clinical Disease Activity indices in patients with psoriatic arthritis: a cross-sectional study. J Rheumatol. 2016;43(9):1749–54. 10.3899/JRHEUM.151491. 27252430 10.3899/JRHEUM.151491

[17] Bello N, Etcheto A, Béal C, Dougados M, Moltó A. Evaluation of the impact of fibromyalgia in disease activity and treatment effect in spondyloarthritis. Arthritis Res Ther. 2016;18(1). 10.1186/S13075-016-0943-Z. 10.1186/s13075-016-0943-z PMC4748456 26860612

[18] Macfarlane GJ, Barnish MS, Pathan E, et al. Co-occurrence and characteristics of patients with Axial Spondyloarthritis who Meet Criteria for Fibromyalgia: results from a UK National Register. Arthritis Rheumatol (Hoboken NJ). 2017;69(11):2144–50. 10.1002/ART.40185. 10.1002/ART.40185 28622461

[19] Baraliakos X, Regel A, Kiltz U, et al. Patients with fibromyalgia rarely fulfil classification criteria for axial spondyloarthritis. Rheumatology (Oxford). 2018;57(9):1541–7. 10.1093/RHEUMATOLOGY/KEX318. 28968885 10.1093/RHEUMATOLOGY/KEX318

[20] Macfarlane GJ, Pathan E, Siebert S, et al. AxSpA patients who also meet criteria for fibromyalgia: identifying distinct patient clusters using data from a UK national register (BSRBR-AS). BMC Rheumatol. 2019;3(1). 10.1186/S41927-019-0066-7. 10.1186/s41927-019-0066-7 PMC6532149 31143851

[21] Moltó A, Etcheto A, Gossec L, et al. Evaluation of the impact of concomitant fibromyalgia on TNF alpha blockers’ effectiveness in axial spondyloarthritis: results of a prospective, multicentre study. Ann Rheum Dis. 2018;77(4):533–40. 10.1136/ANNRHEUMDIS-2017-212378. 29183878 10.1136/ANNRHEUMDIS-2017-212378

[22] Macfarlane GJ, MacDonald RIR, Pathan E, et al. Influence of co-morbid fibromyalgia on disease activity measures and response to tumour necrosis factor inhibitors in axial spondyloarthritis: results from a UK national register. Rheumatology (Oxford). 2018;57(11):1982–90. 10.1093/RHEUMATOLOGY/KEY206. 30053166 10.1093/RHEUMATOLOGY/KEY206 PMC6199528

[23] Wolfe F, Clauw DJ, Fitzcharles MA, et al. 2016 revisions to the 2010/2011 fibromyalgia diagnostic criteria. Semin Arthritis Rheum. 2016;46(3):319–29. 10.1016/J.SEMARTHRIT.2016.08.012. 27916278 10.1016/J.SEMARTHRIT.2016.08.012

[24] Rudwaleit M, Van Der Heijde D, Landewé R, et al. The development of Assessment of SpondyloArthritis international society classification criteria for axial spondyloarthritis (part II): validation and final selection. Ann Rheum Dis. 2009;68(6):777–83. 10.1136/ard.2009.108233. 19297344 10.1136/ard.2009.108233

[25] Taylor W, Gladman D, Helliwell P, Marchesoni A, Mease P, Mielants H. Classification criteria for psoriatic arthritis: development of new criteria from a large international study. Arthritis Rheum. 2006;54(8):2665–73. 10.1002/ART.21972. 16871531 10.1002/ART.21972

[26] Collins SL, Moore RA, McQuay HJ. The visual analogue pain intensity scale: what is moderate pain in millimetres? Pain. 1997;72(1–2):95–7. 10.1016/S0304-3959(97)00005-5. 9272792 10.1016/S0304-3959(97)00005-5

[27] Garrett S, Jenkinson T, Kennedy LG, Whitelock H, Gaisford P, Calin A. A new approach to defining disease status in ankylosing spondylitis: The bath ankylosing spondylitis disease activity index. *J Rheumatol*. 1994;21(12):2286–2291. Accessed June 12, 2021. https://pubmed.ncbi.nlm.nih.gov/7699630/. 7699630

[28] Lukas C, Landewé R, Sieper J, et al. Development of an ASAS-endorsed disease activity score (ASDAS) in patients with ankylosing spondylitis. Ann Rheum Dis. 2009;68(1):18–24. 10.1136/ard.2008.094870. 18625618 10.1136/ard.2008.094870

[29] Calin A, Garrett S, Whitelock H, O’Hea J, Mallorie P, Jenkinson T. A new approach to defining functional ability in ankylosing spondylitis: the development of the bath ankylosing spondylitis functional index. J Rheumatol. 1994;21(12):2281–5. 10.3109/9780203214237-70. 7699629 10.3109/9780203214237-70

[30] Heuft-Dorenbosch L, Spoorenberg A, Van Tubergen A, et al. Assessment of enthesitis in ankylosing spondylitis. Ann Rheum Dis. 2003;62(2):127–32. 10.1136/ARD.62.2.127. 12525381 10.1136/ARD.62.2.127 PMC1754445

[31] Gladman DD, Cook RJ, Schentag C et al. The clinical assessment of patients with psoriatic arthritis: Results of a reliability study of the Spondyloarthritis Research Consortium of Canada. *J Rheumatol*. 2004;31(6):1126–1131. Accessed October 13, 2022. https://pubmed.ncbi.nlm.nih.gov/15170925/. 15170925

[32] Schoels MM, Aletaha D, Alasti F, Smolen JS. Disease activity in psoriatic arthritis (PsA): defining remission and treatment success using the DAPSA score. Ann Rheum Dis. 2016;75(5):811–8. 10.1136/annrheumdis-2015-207507. 26269398 10.1136/annrheumdis-2015-207507

[33] Burckhardt CS, Clark SR, Bennett RM. The fibromyalgia impact questionnaire: development and validation. J Rheumatol. 1991;18(5):728–33. https://pubmed.ncbi.nlm.nih.gov/1865419/. Accessed May 29, 2022. 1865419

[34] Duffield SJ, Miller N, Zhao S, Goodson NJ. Concomitant fibromyalgia complicating chronic inflammatory arthritis: a systematic review and meta-analysis. Rheumatology (Oxford). 2018;57(8):1453–60. 10.1093/RHEUMATOLOGY/KEY112. 29788461 10.1093/RHEUMATOLOGY/KEY112 PMC6055651

[35] Kancharla H, Jain S, Mishra S, et al. Fibromyalgia influences health-related quality of life and disease activity in psoriatic arthritis. Rheumatol Int. 2022;42(3):511–7. 10.1007/S00296-021-04925-0. 34251497 10.1007/S00296-021-04925-0

[36] Marchesoni A, Macchioni P, Gasparini S, et al. Use of Ultrasonography to Discriminate Psoriatic Arthritis from Fibromyalgia: a post-hoc analysis of the ULISSE Study. J Clin Med. 2021;11(1). 10.3390/JCM11010180. 10.3390/jcm11010180 PMC8745640 35011921

[37] Iannone F, Nivuori M, Fornaro M, Venerito V, Cacciapaglia F, Lopalco G. Comorbid fibromyalgia impairs the effectiveness of biologic drugs in patients with psoriatic arthritis. Rheumatology (Oxford). 2020;59(7):1599–606. 10.1093/RHEUMATOLOGY/KEZ505. 31652315 10.1093/RHEUMATOLOGY/KEZ505

[38] Van Der Heijde D, Ramiro S, Landewé R et al. 2016 update of the ASAS-EULAR management recommendations for axial spondyloarthritis. 17. 10.1136/annrheumdis-2016-210770. 10.1136/annrheumdis-2016-210770 28087505

[39] Haliloglu S, Carlioglu A, Akdeniz D, Karaaslan Y, Kosar A. Fibromyalgia in patients with other rheumatic diseases: prevalence and relationship with disease activity. Rheumatol Int. 2014;34(9):1275–80. 10.1007/S00296-014-2972-8. 24589726 10.1007/S00296-014-2972-8

[40] Salaffi F, De Angelis R, Carotti M, Gutierrez M, Sarzi-Puttini P, Atzeni F. Fibromyalgia in patients with axial spondyloarthritis: epidemiological profile and effect on measures of disease activity. Rheumatol Int. 2014;34(8):1103–10. 10.1007/S00296-014-2955-9. 24509896 10.1007/S00296-014-2955-9

[41] Azevedo VF, Paiva E dos, Felippe S, Moreira LRH. RA. Occurrence of fibromyalgia in patients with ankylosing spondylitis. *Rev Bras Reumatol*. 2010;50(6):646–650. Accessed October 13, 2022. https://pubmed.ncbi.nlm.nih.gov/21243305/. 21243305

[42] Navarro-Compán V, Boel A, Boonen A, et al. The ASAS-OMERACT core domain set for axial spondyloarthritis. Semin Arthritis Rheum. 2021;51(6):1342–9. 10.1016/J.SEMARTHRIT.2021.07.021. 34489113 10.1016/J.SEMARTHRIT.2021.07.021

